# Refined Protein–Sugar Interactions in the Martini Force Field

**DOI:** 10.1021/acs.jctc.4c01092

**Published:** 2024-11-08

**Authors:** Maziar Heidari, Mateusz Sikora, Gerhard Hummer

**Affiliations:** † Department of Theoretical Biophysics, Max Planck Institute of Biophysics, Max-von-Laue Straße 3, 60438 Frankfurt am Main, Germany; ‡ Malopolska Centre of Biotechnology, Jagiellonian University, 30-387 Kraków, Poland; ¶ Institute of Biophysics, Goethe University Frankfurt, 60438 Frankfurt am Main, Germany

## Abstract

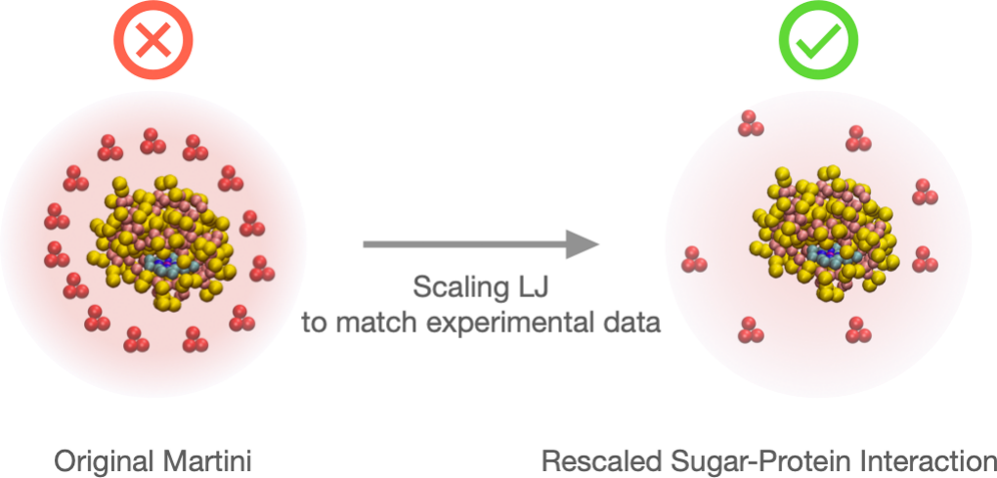

Sugar molecules play important roles as mediators of biomolecular interactions in cellular functions, disease, and infections. Molecular dynamics simulations are an indispensable tool to explore these interactions at the molecular level. The large time and length scales involved frequently necessitate the use of coarse-grained representations, which heavily depend on the parametrization of sugar–protein interactions. Here, we adjust the sugar–protein interactions in the widely used Martini 2.2 force field to reproduce the experimental osmotic second virial coefficients between sugars and proteins. In simulations of two model proteins in glucose solutions with adjusted force field parameters, we observe weak protein–sugar interactions. The sugar molecules are thus acting mainly as crowding agents, in agreement with experimental measurements. The procedure to fine-tune sugar–protein interactions is generally applicable and could also prove useful for atomistic force fields.

## Introduction

In biological systems, proteins interact closely with the many different osmolytes in the surrounding solution,^1^ including amino acids, polyols and various sugars. As crowders, the osmolytes in the intracellular medium affect protein-folding equilibria, protein stability, protein self-assembly and complex formation.^2,3^ In cells, sugars are present not only in solution, but are also covalently attached to many proteins through N- and O-glycosylation processes.^4^ These protein modifications impact the intrinsic kinetics of protein interactions,^5^ protein dynamics^6^ and modulate the liquid–liquid phase separation (LLPS) of intrinsically disordered proteins (IDPs),^7^ including the permeability barrier of the nuclear pore complex.^8,9^

Molecular simulations have been widely used to resolve the details of LLPS in atomistic and coarse-grained simulations. However, protein glycosylation is often ignored, yet glycans can have a substantial impact on the phase behavior and aging.^8^ To accurately capture glycosylation effects, it is crucial to properly balance the energetics of the interactions between sugars, solvents and proteins. Similarly, MD simulations have been used to explore the role of N-glycans in ligand binding,^10^ protein flexibility,^11^ and glycan shielding.^12^ For the Martini force field, which is widely used for coarse-grained simulations of biological systems,^13−16^ recent studies have shown that the protein–protein^17−19^ and osmolyte-osmolyte^20^ interactions have to be rescaled to match experimental observations. In simulations of complex biological systems, it is essential to accurately model cross interactions between components; however, the correct parameters for protein–sugar interactions remain largely unexplored. Here, we address this challenge by taking advantage of detailed osmotic measurements^21,22^ probing the interactions between proteins and glucose, which represents well the typical monosaccharides forming glycans. We match the measured interaction strength in the Martini 2.2 force field by scaling the cross interaction between proteins and glucose. A single scaling factor reproduces the experimentally determined values of the osmotic second virial coefficient for two model systems: cytochrome *c* and α-chymotrypsin dimers in glucose solution. With the “stickiness” of sugar–protein interactions properly adjusted, we then study the organization of glucose molecules around the proteins.

## Methods

We used the Martini 2.2 coarse-grained (CG) model^13^ to describe the energetics. We tuned the Lennard-Jones (LJ) interactions between sugar and proteins by adapting the procedure used previously to scale interactions between proteins^17,18^ and polysaccharides.^20^ Specifically, we rescaled the protein–sugar LJ interaction strengths ϵ with a λ parameter by setting ϵ_λ_ = ϵ_0_ + λ(ϵ_original_ – ϵ_0_). For λ = 1, the original cross interaction strength ϵ_original_ between sugars and proteins in the Martini model is recovered. For λ = 0, one obtains a very weak strength of ϵ_0_ = 2 kJ/mol corresponding to repulsive interactions. The scaling parameter λ is adjusted to reproduce the measured dependence of the osmotic second virial coefficient of a protein on the glucose concentration in the aqueous solution. We then validate λ by comparing the predicted effect of glucose on the dimerization equilibrium for a different protein to experiments.

We computed the osmotic second virial coefficients from the integrated radial distribution function (RDF) of glucose around the protein. McMillan-Mayer theory^23^ relates the osmotic second virial coefficient *B*_*ij*_ for solutes *i* and *j* to their RDF *g*_*ij*_(*r*) integrated over the distance *r*
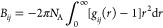
1with *N*_A_ being the Avogadro constant. Formally, the integral in eq 1, converges in the limit of large systems and in a grand canonical ensemble.^24^ For finite systems, however, radial distribution functions *g*_*ij*_ (*r*) can noticeably deviate from one,^24,25^ which will lead to a cubically growing divergence of *B*_*ij*_. To address these challenges, we used systems of comparably large size and compared the results obtained for different sizes. In addition, we explicitly corrected for any remaining small deviations of the RDF from one at large distances. For this, we averaged the RDF tail over a distance window of width Δ
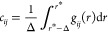
2with r* being a distance larger than the correlation length. We then replaced the ideal RDF large-distance limit of one in eq 1 by this average to estimate the osmotic second virial coefficient *B*_*ij*_ as
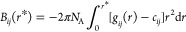
3We compared results for *B*_*ij*_(r*) obtained for large integration cutoffs r* to experimental values of protein-glucose cross second virial coefficients (*B*_23_) and glucose-dependent protein–protein dimerization constants obtained by Pielak and co-workers.^21,22^

### Dimer Dissociation

We estimate the rate of α-chymotrypsin dimer dissociation from the statistics of dissociation events in multiple simulation runs starting from a dimerized state. For this, we use a maximum-likelihood estimator
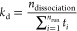
4where *n*_dissociation_ ≤ *n*_run_ is the number of dissociation events observed in the *n*_run_ runs and *t*_*i*_ is the time point of the event or, if no dissociation occurred, the duration of the run. The denominator is thus the aggregate time in the bound state across the different runs.

## Simulation Models

As model systems, we used the structures of *Saccharomyces cerevisiae* iso-1 cytochrome *c* (PDB ID: 2ORL;^26^ nonheme ligands removed) and *Bos taurus* α-chymotrypsin dimer (PDB ID: 4CHA(27)). We used the martinize.py script^14,28^ to convert the protein structures into coarse-grained representation within the Martini 2.2 scheme.^13^ We used the DSSP algorithm to assign the secondary structure restraints.^29^ We used Elastic Network in Dynamics (ElNeDyn) to generate elastic bonds within the ordered domains to maintain the tertiary structure,^30^ with default parameter settings, a distance range of 0.9 nm and a force constant of 500 kJ/(mol nm^2^). All systems were solvated with coarse-grained water containing 10% antifreeze (WF) particles.^13^ Charge neutrality and salt molarity were established by replacing water particles with ions following standard GROMACS procedure. We used Cl^–^ ions to neutralize the cytochrome *c* systems so as to simulate the experimental deionized water condition.^21^ We added Na^+^ and Cl^–^ ions to match the experimental concentration of 200 mM NaCl^22^ in overall neutral α-chymotrypsin systems. We used the GROMACS 2020.6 software package to simulate all systems^31^ and prepared visualizations using the Visual Molecular Dynamics (VMD) software.^32^

Cytochrome *c* binds heme C within a dedicated pocket. With heme B parameters available in the Martini force field, we used heme B instead of heme C to construct the holo state of the protein.^33^ In heme C, the two vinyl groups of heme B are replaced by thioether linkages, which covalently attach the heme to two cysteine residues of cytochrome *c*. Being largely buried within the near-rigid protein scaffold, the differences between the two hemes are negligible for the purpose of our calculations. To mimic the low experimental pH of 3.5 of the α-chymotrypsin dimer solution,^22^ we used PROPKA^34,35^ with the all-atom AMBER ff99 energy function^36^ and the CHARMM-GUI server^37,38^ to determine amino acid protonation states. We found that six amino acids were protonated in each monomer (Figure S1). We modified the protonated residues by adjusting their charge accordingly and changing the type of the side chain particle in the Martini force field, i.e., from Qa to P3 for ASP, from Qa to P1 for GLU and from P1 to Qd for HIS.

**Figure 1:**
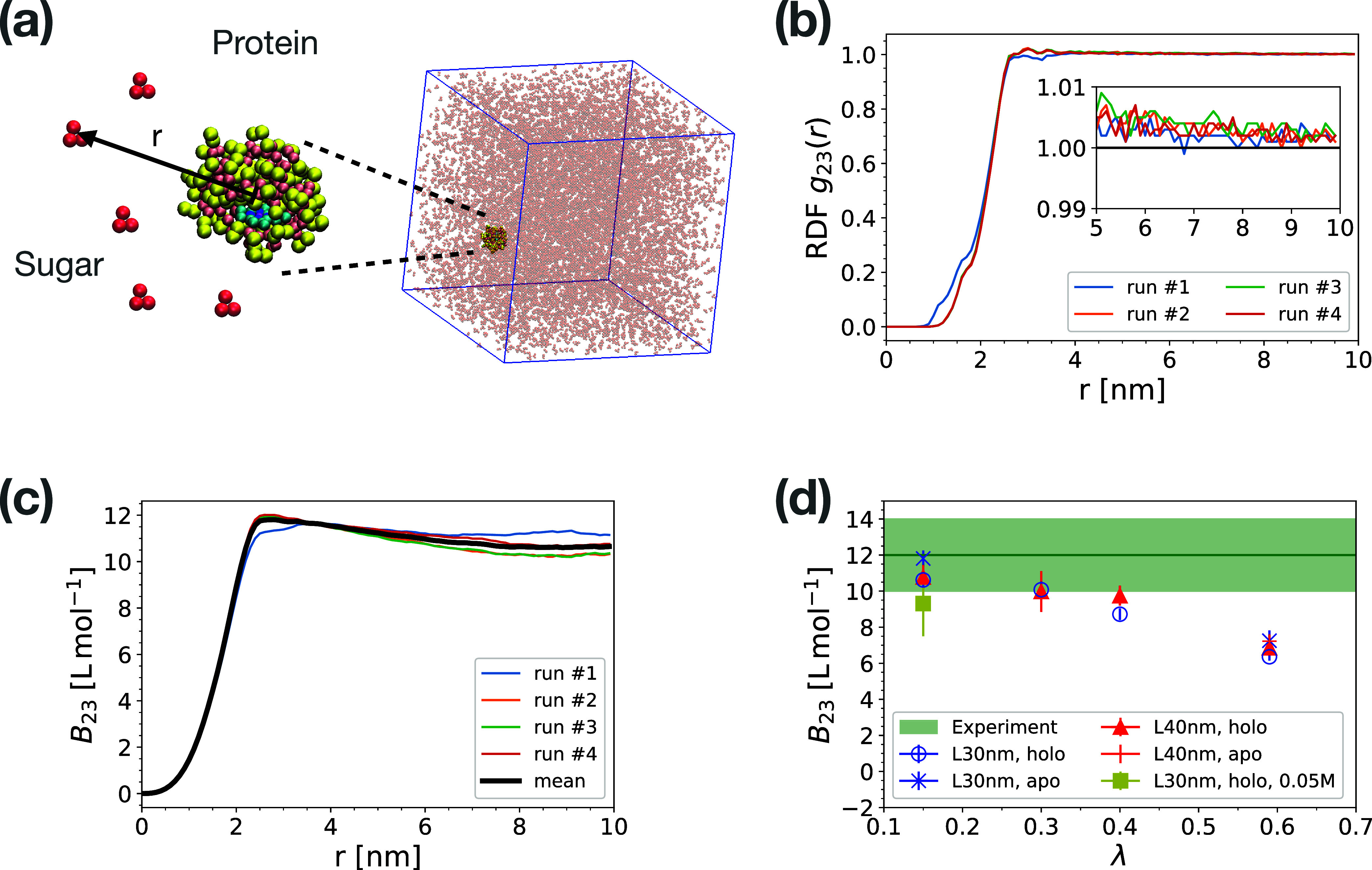
Balancing protein and sugar interactions. (a) Coarse-grained model of glucose (red beads) and cytochrome *c* in holo state (yellow and pink beads). The heme C molecule is shown in the pocket of cytochrome (cyan). The simulation box size is 30 nm and the glucose concentration is 0.5 M. (b) Radial distribution function of glucose molecules about the cytochrome *c* protein for scale factors γ = 0.5 and λ = 0.15. The distance is measured between the centers of mass of the two components. The results of four independent runs are shown with different colors. The inset zooms in on the convergence region of RDFs within the range of 5 to 10 nm. (c) Osmotic second virial coefficient of protein–sugar interaction calculated by integrating RDFs in panel (b) according to eq 3 for λ = 0.15. (d) Osmotic second virial coefficient of cytochrome *c* and glucose as a function of cross interaction scaling parameter λ. The green shaded area shows the experimental value^21^ with standard error. The symbols in panel (d) show averages of *B*_23_(r*) over the range 8 nm < r* < 10 nm (eqs 2 and 3) and across the different runs, with error bars indicating standard errors of the mean. For λ = 0.15, also a system with 0.05 M glucose was studied (filled square) instead of the 0.5 M in the other systems. Results are shown for holo (circle, triangle, square symbols) and apo cytochrome *c* (crosses) simulated in boxes of size *L* ≈ 30 nm (blue) and 40 nm (red).

We performed energy minimization on the initial models using steepest descent with an energy minimization step of 0.01 nm and a force tolerance of 1000 kJ mol^–1^ nm^–1^. This was followed by 150 ns of MD simulation with a time step of 0.015 ps at a temperature of 300 K established using a velocity rescaling thermostat^39^ with a time constant of 1 ps. Then we performed 750 ns MD simulation in an isothermal–isobaric ensemble at a temperature of 300 K established using a velocity rescaling thermostat and a pressure of 1 bar established using an isotropic Berendsen barostat^40^ with time constant 12 ps and compressibility 3 × 10^–4^ bar^–1^. For production runs, we used a velocity rescaling thermostat and an isotropic Parrinello–Rahman barostat^41^ with identical time constants. The time step of production runs was set to 0.03 ps. The production runs were at least 60 μs long for glucose in solution and 30 μs for all other cases. In all simulations we used cubic boxes of size *L* ≈ 30 nm or 40 nm. The concentrations of glucose for cytochrome *c* and α-chymotrypsin systems were set to 0.5 M (cytochrome *c*; Figure 1a) and 0.1 M (α-chymotrypsin monomer and dimer; Figure 3a), respectively. For cytochrome *c*, we also performed simulations with 0.05 M glucose. The RDFs were computed between the centers of mass of protein and sugar molecules using the GROMACS built-in command *gmx rdf*. The bin size, maximum range and configurational sampling frequency of RDFs were set to 0.1 nm, 10 nm and 1.5 ns, respectively. The RDFs were orientationally averaged about the proteins. Previous studies have introduced optimum Lennard-Jones scaling parameters α = 0.3 (ref (17)) and γ = 0.5 (ref (20)) for protein–protein and sugar–sugar interactions, respectively. Here, we kept the prescribed sugar–sugar interaction scaling, but consistently used α = 0.7 scaling for the optimal protein–protein interaction as determined before.^18^ The sugar–sugar interaction scaling was validated against earlier findings by comparing the values of *B*_22_ at different γ-scaling (see Figures S2 and S3). To match the osmotic measurements,^21,22^ we varied the interaction scaling factor λ of protein–sugar interactions. For the RDF tail correction of sugar–sugar interactions, we used Δ = 1 nm and r* = 5 nm; and for the sugar interactions with the proteins cytochrome *c* and α-chymotrypsin, we used Δ = 2 nm and r* = 10 nm.

## Results

### Osmotic Second Virial Coefficient for the Glucose–Cytochrome *c* Interaction

To determine the preference of the glucose molecules to bind to cytochrome *c*, we first analyzed the RDFs of glucose around cytochrome *c*. In Figure 1b we show examples of four independent simulation runs at λ = 0.15. We found that the excluded radius extends approximately 1 nm from the protein center. Between 3 and 4 nm, close to the protein surface, the glucose molecules form a very weakly structured layer. Beyond, the RDF approaches the ideal gas limit of one. The inset zooms in on the convergence of RDFs, highlighting the small but noticeable deviations from one, as estimated by *c*_*ij*_ in eq 2. Similar RDFs are found for λ = 0.3 (Figure S4c,d), with the structured layers enhanced for larger λ values (Figure S4e–h). The cross osmotic second virial coefficient *B*_23_ for glucose and protein calculated from different simulation runs converge at distances of r* ≈ 8 to 10 nm (Figure 1c). To find the optimal value of λ corresponding to the experimentally measured values of *B*_23_, we quantified the dependence of *B*_23_ on λ (Figure 1d). A monotonic decrease of *B*_23_ with increasing λ is consistent with the rise in attractive interactions between protein and sugar. The calculated *B*_23_ (λ = 0.15) = 10.63 ± 0.33 L mol^–1^ matches the experimental value of *B*_23_^exp^ = 12 ± 2 L mol^–1^,^21^ independent of the simulation box size (compare filled circles and triangles in Figure 1d). To test how the results depend on the presence of the heme ligand, we repeated simulations with heme B removed from the cytochrome *c* pocket (apo state). This led to a very small change in *B*_23_ (star symbols in Figure 1d) compared with the holo state. As shown in Figure S6, in the apo state glucose molecules can partially penetrate the binding pocket, leading to a higher first peak in the RDF. As a test of a possible glucose concentration dependence, the *B*_23_ value obtained in simulations with a reduced glucose concentration of 0.05 M is statistically consistent with the results obtained for 0.5 M glucose (Figures 1d and S7). In summary, after scaling the glucose–protein interactions with a factor of λ = 0.15, we obtained a *B*_23_ value in agreement with measurements on cytochrome *c*. For higher values, λ ≥ 0.4, the calculated *B*_23_ is noticeably too small.

**Figure 2:**
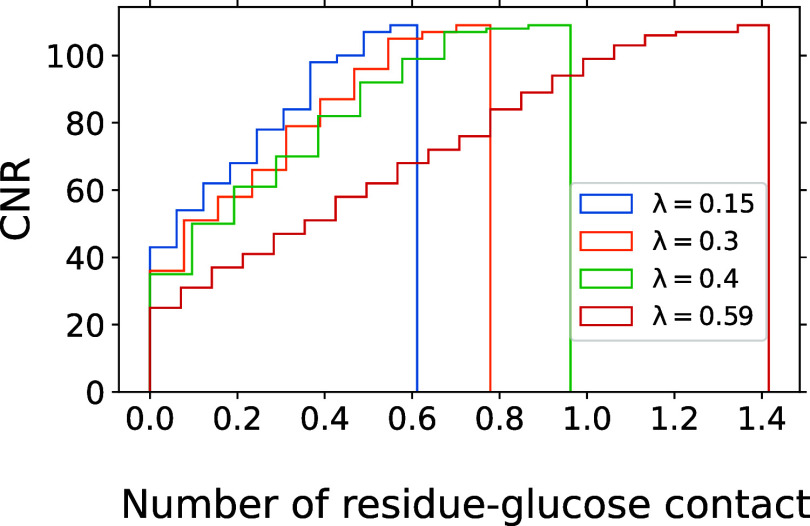
Glucose–protein contact number distribution for holo cytochrome *c* in 0.5 M glucose solution. Shown is the cumulative number of residues (CNR) as a function of their mean number of contacting glucose beads, i.e., the number of amino acids that have fewer residue-glucose contacts on average than the number specified on the *x* axis. A glucose molecule is considered to be in contact with a residue if any glucose bead is within 0.94 nm of the residue center of mass. The steps at zero reflect the fact that for λ = 0.15, 0.3, 0.4 and 0.59, there are 6, 11, 15, and 19 residues without any glucose contacts during the simulations, respectively. The CNRs were obtained using the average number of contacts across four independent simulation runs.

Having obtained an optimal scaling parameter λ = 0.15 for the sugar–protein interactions in Martini 2.2, we next set out to determine how the relatively weak sugar–protein interactions govern the arrangement of glucose molecules in the vicinity of the two proteins. We calculated the cumulative distribution of the average number of contacts between glucose molecules and the residues of cytochrome *c* as shown in Figure 2. Consistent with the described *B*_23_ dependence on λ, we saw only very transient contacts that noticeably decrease in number as λ is reduced.

### Glucose Effect on α-Chymotrypsin Dimerization

**Figure 3:**
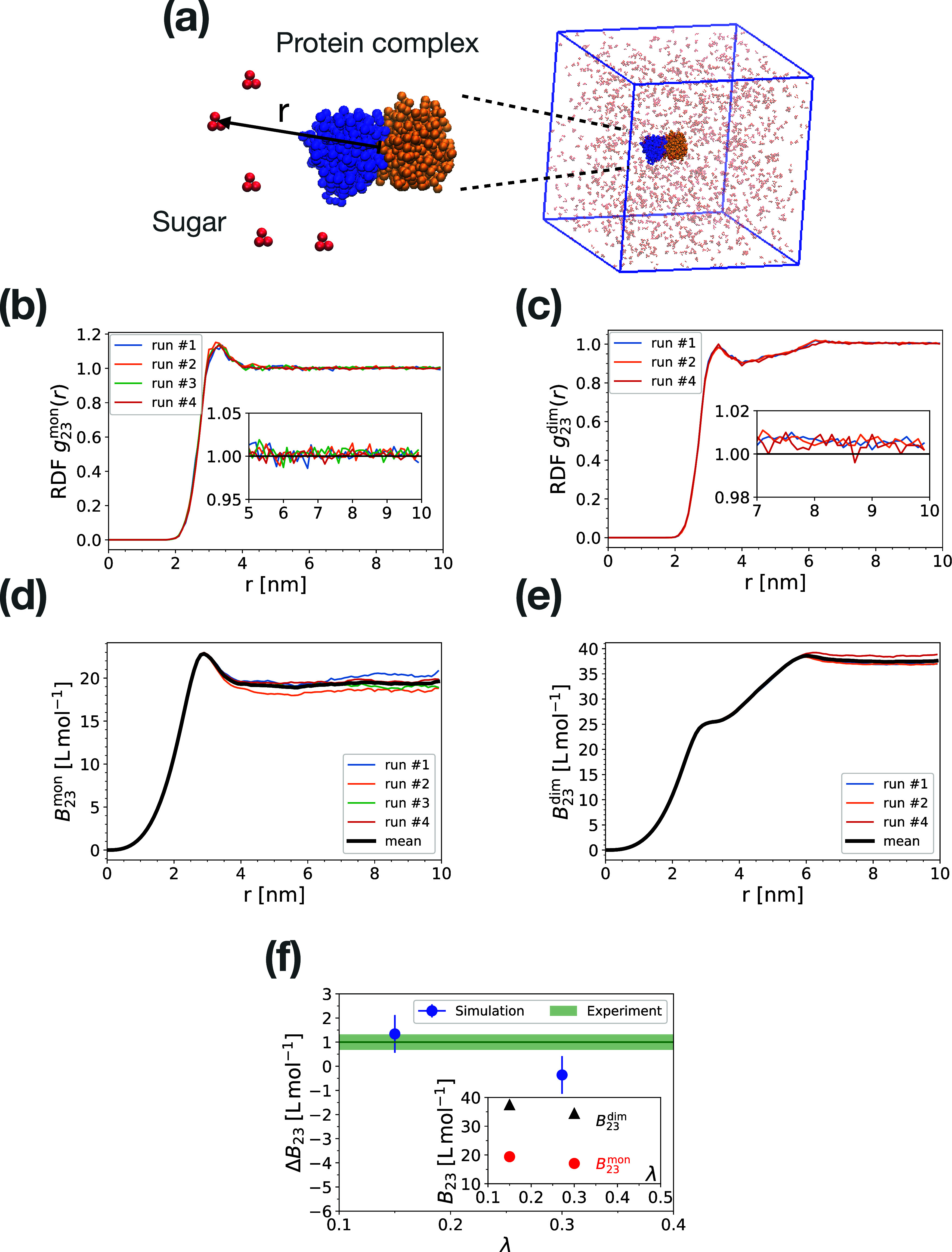
Balancing protein and sugar interactions. (a) Coarse-grained model of glucose (red beads) and α-chymotrypsin dimer (PDB ID: 4CHA) with monomers colored in blue and orange. The simulation box size is 30 nm and the glucose concentration is 0.1 M. (b, c) Radial distribution function of glucose molecules about α-chymotrypsin monomer and dimer with scale factors α = 0.7, γ = 0.5 and λ = 0.15 for protein–protein, sugar–sugar and protein–sugar interactions, respectively. The distance is measured between the centers of mass of the components. The results of independent runs are shown with different colors. Insets zoom in on the convergence region of RDFs within the range of 5 to 10 nm. (d, e) Convergence of osmotic second virial coefficients of sugar and protein in monomer (*B*_23_^mon^) and dimer (*B*_23_^dim^) configurations obtained by integrating the RDFs of panels (b) and (c) according to eq 3. (f) Effect of glucose on the stability of α-chymotrypsin dimer, Δ*B*_23_ = 2*B*_23_^mon^ – *B*_23_^dim^, as a function of protein–sugar interaction scaling parameter λ. The green shaded area shows the experimental value ± SE,^22^ which matches with the simulation results at λ = 0.15. The inset shows the osmotic second virial coefficients of sugar and α-chymotrypsin in monomer and dimer states. In dimeric α-chymotrypsin, the result of simulation run # 3 is not shown as the dimer dissociated near the beginning of the run (Figure S8a). The symbols and error bars in panel (f) are the average and standard error of means obtained by averaging Δ*B*_23_(*r*) over the range 8 nm < *r* < 10 nm for each run.

To validate the scale factor λ = 0.15, we turned to our second model system, α-chymotrypsin. For this protein, the effect of added glucose on the monomer–dimer equilibrium has been characterized by analytical ultracentrifugation.^22^ The apparent dissociation constant *K*_d_(*C*) depends on sucrose concentrations *C* as *K*_d_(*C*) ≈ *K*_d_^0^ exp(−Δ*B*_23_*C*) with Δ*B*_23_ = 2*B*_23_^mon^ – *B*_23_^dim^ the difference in glucose–protein osmotic second virial coefficients for two protein monomers and a dimer. The experiments found a stabilizing effect for glucose^22^ with Δ*B*_23_ ≈ 1.0 ± 0.3 M^–1^. To calculate this effect for our Martini model with scaled sugar–protein interactions, we first computed the RDF of glucose and the protein in monomer (*B*_23_^mon^) and dimer (*B*_23_^dim^) states separately, as shown in Figure 3b,c. During the simulations, we observed several events of spontaneous dissociation of α-chymotrypsin dimers (Figure S8). Thus, for the computation of RDFs in the dimer state, we considered only the initial time interval during which the monomers were associated. In contrast to the RDFs of cytochrome *c* at λ = 0.15, we found that glucose molecules are more attracted to the α-chymotrypsin monomer (see more prominent peak at *r* = 3 nm in Figure 3b). The decrease in the RDF of glucose around the dimer complex close to *r* = 4 nm can be explained by the asphericity of the dimer (Figure 3c). The RDFs of monomer and dimer at λ = 0.3 also show similar trends (Figure S9). The effect of glucose on the stability of the α-chymotrypsin dimer can be quantified and compared with experimental values^22^ using Δ*B*_23_ = 2*B*_23_^mon^ – *B*_23_^dim^. We find reasonable convergence of *B*_23_ at *r* above 5 and 7 nm for monomer and dimer, respectively (Figure 3d,e). Similar to cytochrome *c*, *B*_23_ decreases with λ for both monomer and dimer (see inset in Figure 3f). Reassuringly, Δ*B*_23_ for λ = 0.15 matches the experimental value for α-chymotrypsin (Figure 3f).

### Dimer Dissociation

In our MD simulations, we observed two α-chymotrypsin dimer dissociation events at times *t*_1_ = 3 μs and *t*_2_ = 17 μs (Figure S7a). In the other two runs, the dimer stayed bound for the duration of the simulations, *t*_3_ = 46 μs and *t*_4_ = 45 μs. The maximum-likelihood estimate for the dissociation rate, eq 4, is *k*_d_ ≈ 1/(55.5 μs). The measured value^22^ of the dimer dissociation constant is approximately *K*_d_ ≈ 1/*K*_2,app_ ≈ 20 μM. If we assume diffusion-limited association, this value of *K*_d_ would correspond to an experimental dissociation rate of approximately *k*_d_ ≈ *K*_d_ × 10^9^ M^–1^ s^–1^ ≈1/(50 μs). Clearly, this near-perfect agreement is somewhat fortuitous, considering the approximations involved and the use of a coarse-grained simulation model. Nevertheless, it is reassuring that also the protein–protein interaction comes out about right for the scale factor α = 0.7 used here.

## Conclusions

We developed an approach to accurately model sugar–protein interactions in the Martini coarse-grained scheme by scaling the cross interaction strength between sugar and protein beads. We determined an optimal scale factor λ = 0.15 by matching the calculated and measured^21^ osmotic second virial coefficients for glucose and cytochrome *c*. With this parameter, we then recovered the effect of glucose on the dimerization equilibrium of α-chymotrypsin.^22^ The small value of the scaling parameter implies a very weak interaction between the sugar and protein, which is in-line with the general picture that the sugar molecules act as inert crowders and interact only weakly with proteins. We expect this effect to be important also for larger saccharides and glycans, as the osmotic coefficient is expected to grow with the degree of polymerization.^20,42^ The weak attractions between sugars and proteins contribute to the inhibitory effect of glycans on protein–protein interactions, e.g., to create a “glycan shield” around antigens that protect against antibody binding.^43^

As a general issue, we conclude that simple mixing rules such as Lorentz–Berthelot, should not be expected to give accurate renderings of binding and phase behavior. Even if AA and BB interactions between two compounds A and B are well balanced (here, proteins and sugars in aqueous solution), there is no guarantee that a predefined mixing scheme will be sufficiently accurate for the AB interactions. We showed that by simply using the geometric average of protein–protein (α ≈ 0.7; ref (18)) and sugar–sugar scale factors (γ = 0.5; ref (20)) for protein–sugar interactions, , the sugar molecules are too sticky and adhere to the protein surface. Even with a lower value of α = 0.3 for protein–protein interactions,^17,20^ the sugar–protein scale factor expected from mixing, λ ≈ 0.39, is larger than our optimal value of λ = 0.15.

Here, we focused on the Martini 2.2 scheme; however, we expect similar reasoning can be made for Martini 3. As the Martini force field has paved the way for simulations of large length-scale and long time-scale problems, we believe that our scaling of sugar–protein interaction is a critical step on the path to whole-cell simulations^44^ as many cellular components interact either with the solution glycans or with post-translationally glycosylated components.

## Supporting Information Available

Figure S1: Protonated residues of α-chymotrypsin; Figure S2: Glucose-glucose radial distribution function and convergence of osmotic second virial coefficient; Figure S3: Osmotic second virial coefficient of glucose-glucose interaction; Figure S4: Radial distribution function of glucose; Figure S5: Convergence of osmotic second virial coefficient; Figure S6: Radial distribution function of glucose around apo cytochrome *c*; Figure S7: Radial distribution function of glucose at a glucose concentration of 0.05 M around holo cytochrome *c*; Figure S8: Inter-monomer distance of two α-chymotrypsin proteins; Figure S9: Radial distribution function of glucose around monomer and dimer α-chymotrypsin (PDF)
