# Supplementary material for: Refined Protein–Sugar Interactions in the Martini Force Field
Source: J Chem Theory Comput. 2024 Nov 8;20(22):10259–65. doi: 10.1021/acs.jctc.4c01092 (PMC11603599; doi:10.1021/acs.jctc.4c01092)
Supplement: Supplementary file 1 — ct4c01092_si_001.pdf [file ct4c01092_si_001.pdf]

Supporting Information: Refined Protein-Sugar Interactions in Martini Force Field

Maziar Heidari,[†] Mateusz Sikora,[‡] and Gerhard Hummer[*,†,¶]

†*Department of Theoretical Biophysics, Max Planck Institute of Biophysics, Max-von-Laue Straße 3, 60438, Frankfurt am Main, Germany*

‡*Malopolska Centre of Biotechnology, Jagiellonian University, 30-387 Kraków, Poland*

¶*Institute of Biophysics, Goethe University Frankfurt, 60438 Frankfurt am Main, Germany*

E-mail: Gerhard.Hummer@biophys.mpg.de

Contents

1 Supporting Figures S1–S9 **S2**

References **S10**

1 Supporting Figures

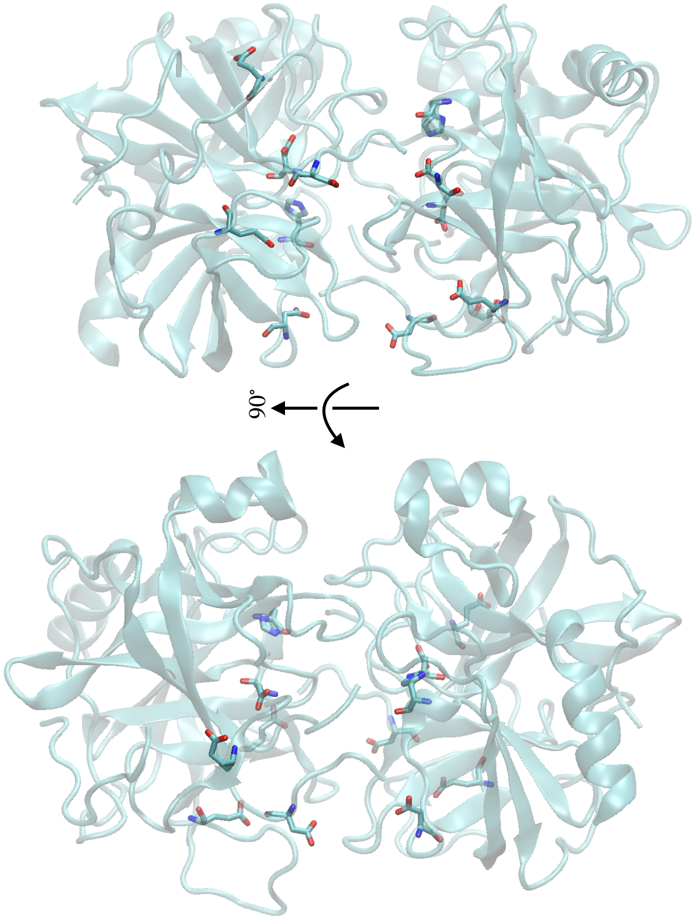

Figure S1: Protonated residues of α-chymotrypsin at pH 3.9 identified using PROPKA[S1,S2] with all-atom AMBER ff99[S3] and CHARMM-GUI server.[S4,S5] In each monomer, GLU20 (pKa = 5.48), HIS57 (pKa = 8.37), ASP64 (pKa = 4.67), GLU70 (pKa = 6.91), ASP153 (pKa = 5.16), and ASP194 (pKa = 3.98) were protonated. The protonated residues are shown as liquorice, and the protein dimer is shown as cartoon. The protonated residues are concentrated at the interface between the monomers.

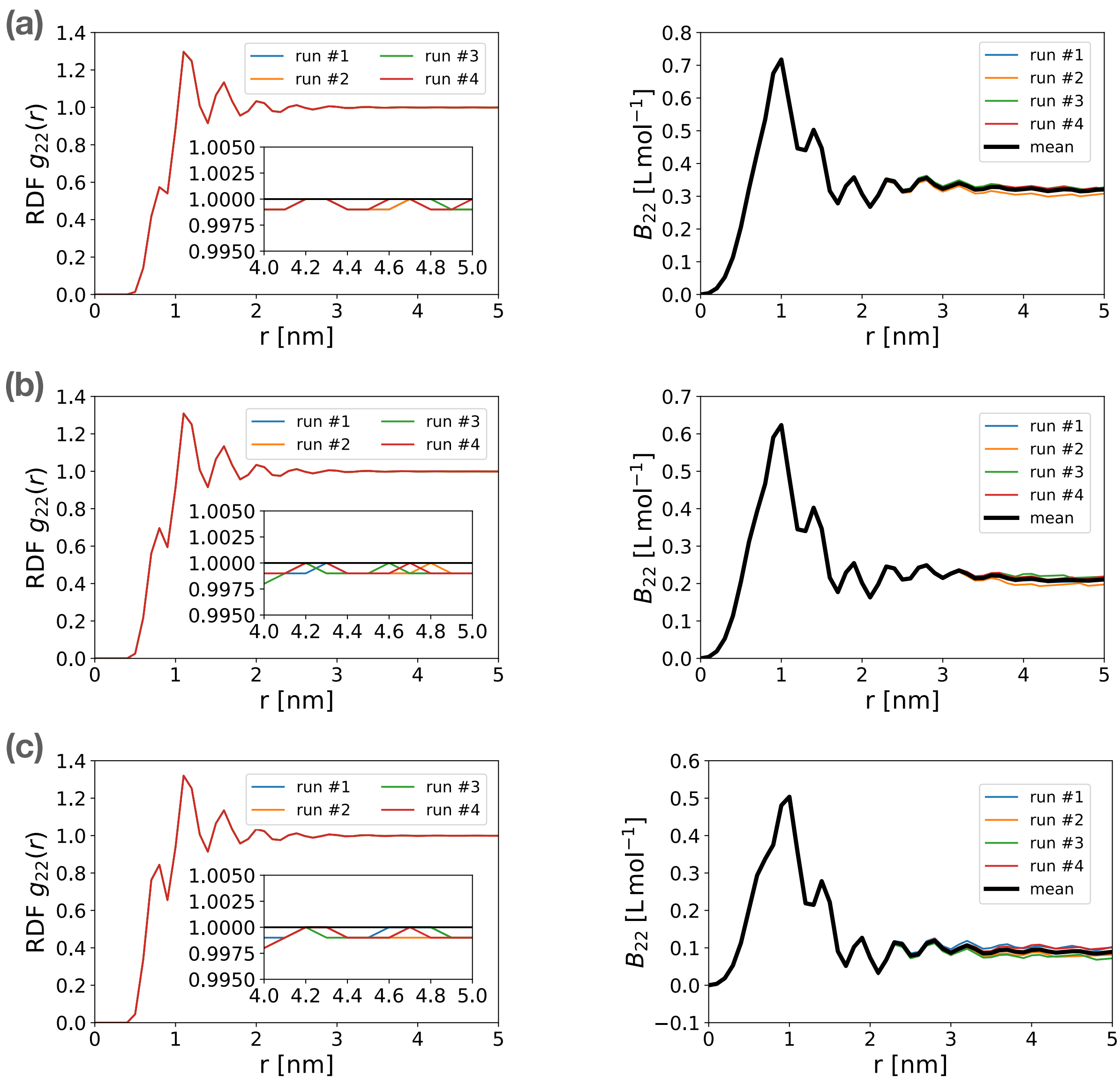

Figure S2: Glucose-glucose radial distribution function (RDF) and convergence of osmotic second virial coefficient. RDFs were computed between the respective centers of mass. In each panel, the RDF (left) and B_{22} (right) of four independent runs are shown. The scaling parameter between sugar-sugar interaction (γ) is $\gamma = 0.4$ (a), $\gamma = 0.5$ (b) and $\gamma = 0.6$ (c). The concentration of the sugar molecules is 0.1 M and the system is a cube of size $L \approx 30$ nm. The inset zooms in on the RDF tails. To compute B_{22}, we used $\Delta = 1$ nm and $r^* = 5$ nm.

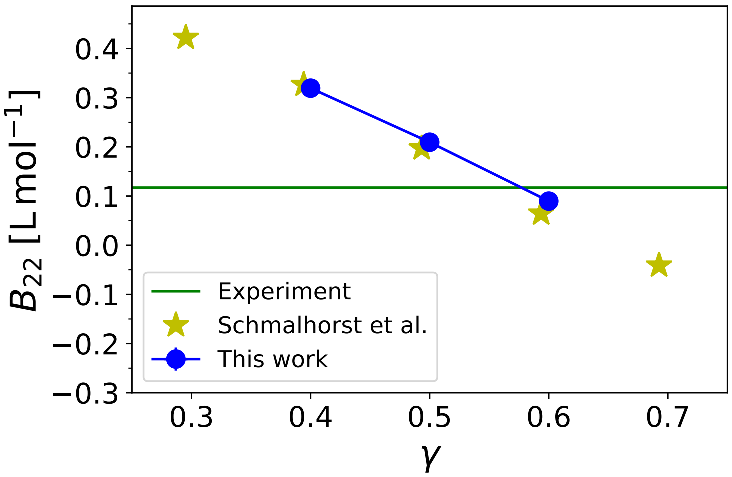

Figure S3: Osmotic second virial coefficient of glucose-glucose interaction. B_{22} is shown against scaling parameter γ using RDFs at concentrations 0.1M of glucose solutions. The results of Schmalhorst et al.[S6] are shown by yellow stars.

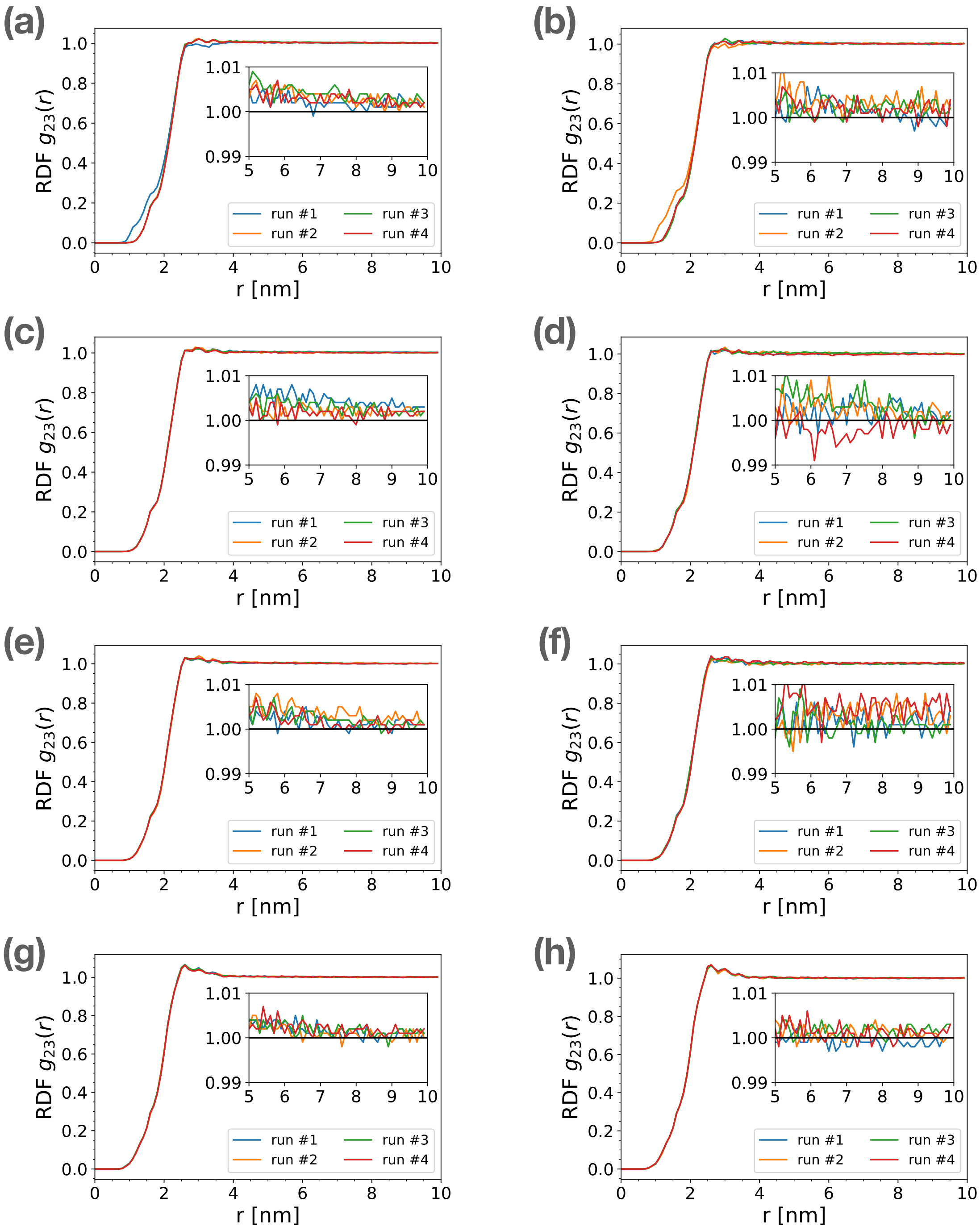

Figure S4: Radial distribution function (RDF) of glucose around holo cytochrome c at a glucose concentration of 0.5 M. The RDFs were computed using different protein-sugar scaling parameters (λ) and simulation box sizes (L). (a, b) $\lambda = 0.15$; (c, d) $\lambda = 0.3$; (e, f) $\lambda = 0.4$; (g, h) $\lambda = 0.59$; (a, c, e, g) $L = 30$ nm; (b, d, f, h) $L = 40$ nm. Insets zoom in on the RDF tail in the range of 5 to 10 nm.

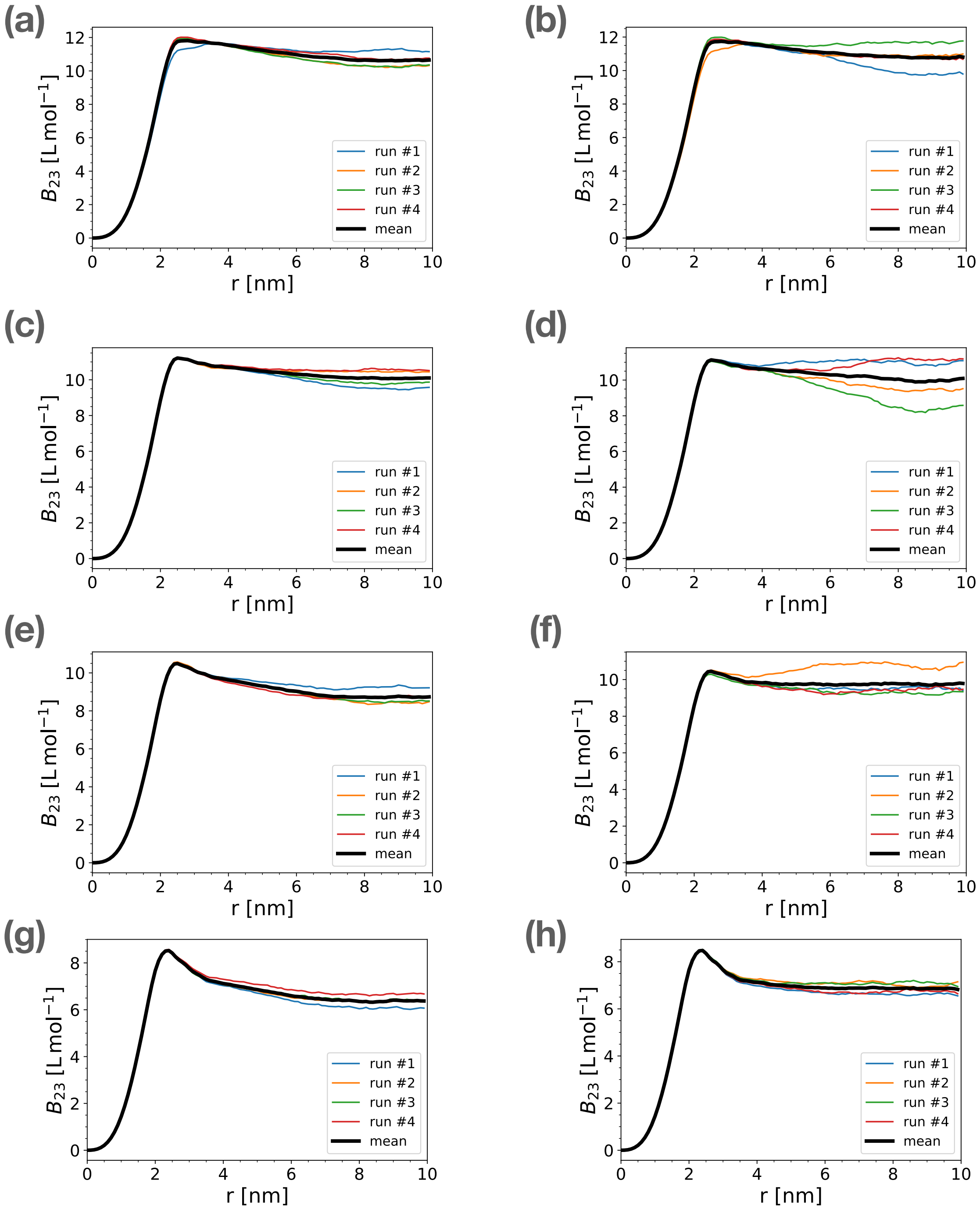

Figure S5: Convergence of osmotic second virial coefficient (B_{23}) for the interaction between glucose and holo cytochrome c at a glucose concentration of 0.5 M. The B_{23} were computed using different protein-sugar scaling parameters (λ) and simulation box sizes (L). (a, b) $\lambda = 0.15$; (c, d) $\lambda = 0.3$; (e, f) $\lambda = 0.4$; (g, h) $\lambda = 0.59$; (a, c, e, g) $L = 30$ nm; (b, d, f, h) $L = 40$ nm. Each panel shows the results of four independent simulation runs (thin lines) and their average (thick black line). To compute B_{23}, we used $\Delta = 2$ nm and $r^* = 10$ nm.

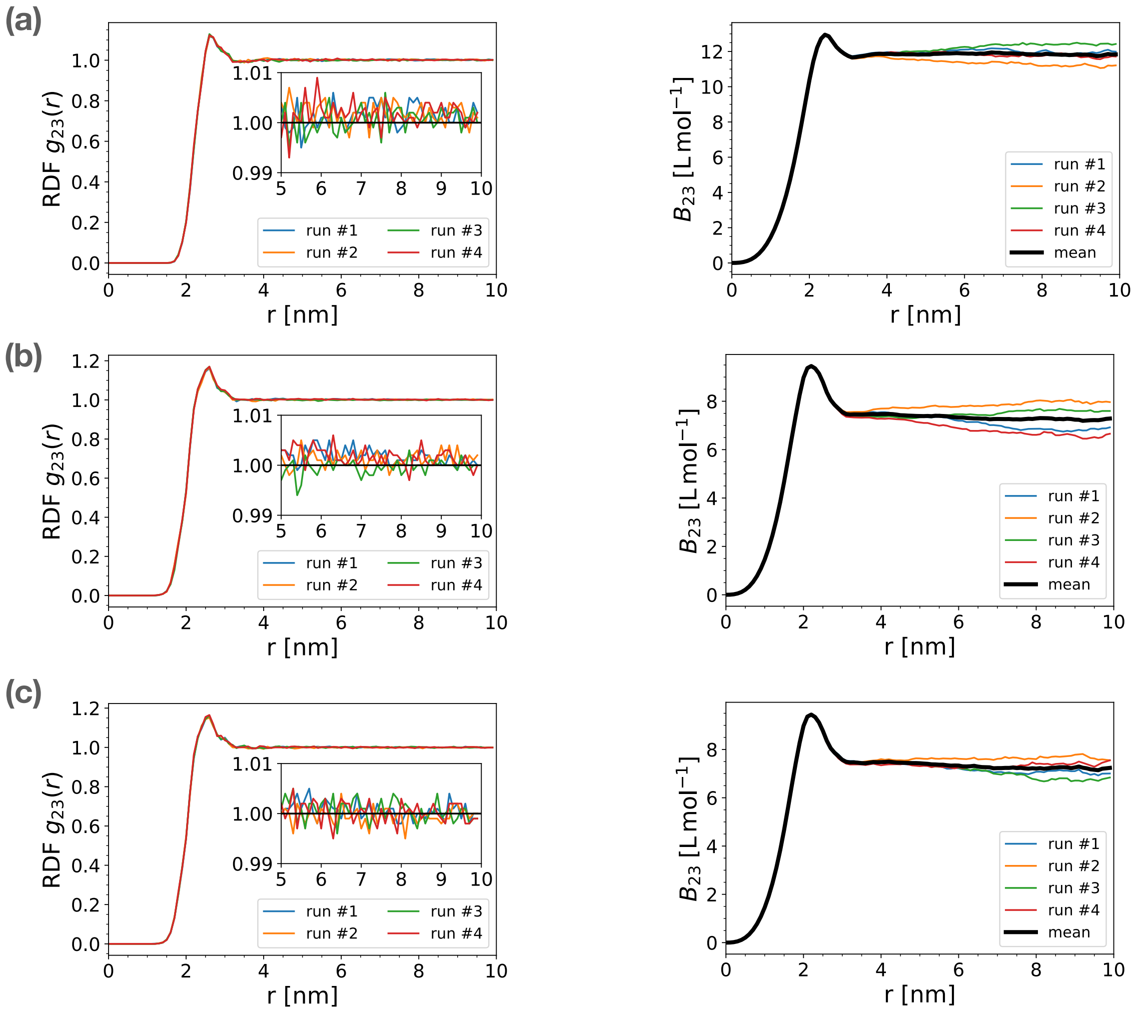

Figure S6: Radial distribution function (RDF) of glucose around apo cytochrome c at a glucose concentration of 0.5 M, and convergence of the associated osmotic second virial coefficient (B_{23}). The RDFs (left column) and B_{23} (right column) were computed using protein-sugar scaling parameter ($\lambda = 0.15$) and simulation box size $L = 30$ nm (a) and ($\lambda = 0.59$) and simulation box size $L = 30$ nm (b) and $L = 40$ nm (c). Insets zoom in on RDF convergence in the range 5 to 10 nm. To compute B_{23}, we used $\Delta = 2$ nm and $r^* = 10$ nm.

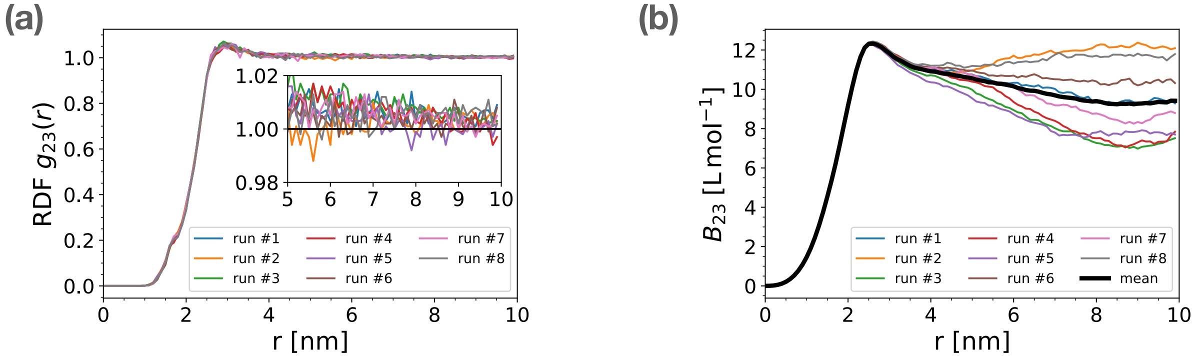

Figure S7: Radial distribution function (RDF) of glucose at a glucose concentration of 0.05 M around holo cytochrome c, and convergence of the associated osmotic second virial coefficient (B_{23}). The RDFs (a) and B_{23} (b) were computed using a protein-sugar scaling parameter $\lambda = 0.15$ and a simulation box size of $L = 30$ nm. Insets zoom in on RDF convergence in the range 5 to 10 nm. To compute B_{23}, we used $\Delta = 2$ nm and $r^* = 10$ nm.

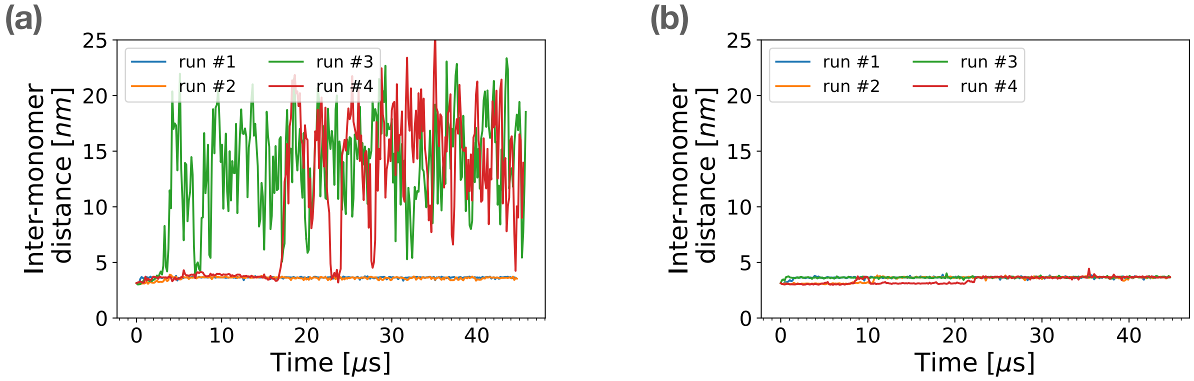

Figure S8: Inter-monomer distance of two α-chymotrypsin proteins starting from a dimeric state. The time series of the distance between monomer centers of geometry in dimer α-chymotrypsin is plotted for $\lambda = 0.15$ (a) and $\lambda = 0.3$ (b). Protein-protein interactions were scaled with $\alpha = 0.7$ (ref S7). The glucose concentration was 0.1 M.

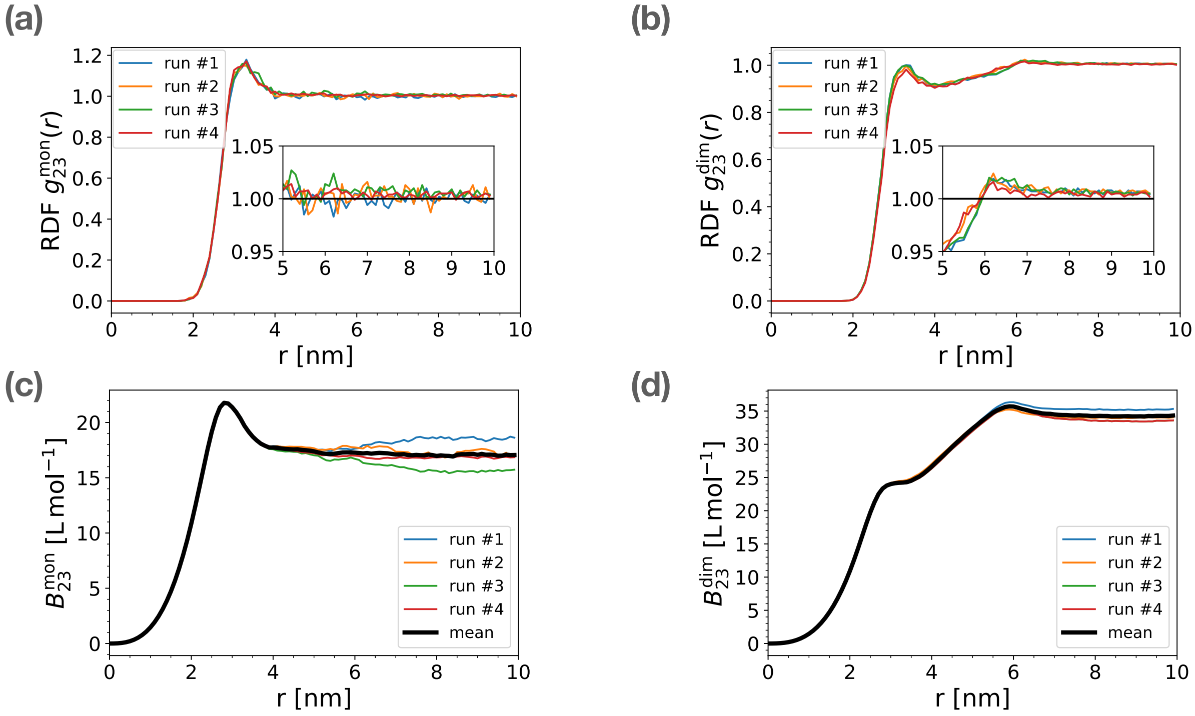

Figure S9: Radial distribution function (RDF) of glucose around monomer and dimer α-chymotrypsin and convergence of the associated osmotic second virial coefficient (B_{23}). The RDFs and B_{23} were computed using a protein-sugar scaling parameter $\lambda = 0.3$ for monomer α-chymotrypsin (a, c) and dimer α-chymotrypsin (b, d). Insets in panels a and b zoom in on the RDF tail in the range of 5 to 10 nm. Each panel shows the results of four independent simulation runs (thin lines). Panels c and show the respective average as thick black line. To compute B_{23}^{mon} and B_{23}^{dim}, we used $\Delta = 2$ nm and $r^* = 10$ nm.

References

(S1) Dolinsky, T. J.; Czodrowski, P.; Li, H.; Nielsen, J. E.; Jensen, J. H.; Klebe, G.; Baker, N. A. PDB2PQR: expanding and upgrading automated preparation of biomolecular structures for molecular simulations. *Nucleic Acids Res.* **2007**, *35*, W522–W525.

(S2) Jurrus, E.; Engel, D.; Star, K.; Monson, K.; Brandi, J.; Felberg, L. E.; Brookes, D. H.; Wilson, L.; Chen, J.; Liles, K., et al. Improvements to the APBS biomolecular solvation software suite. *Protein Sci.* **2018**, *27*, 112–128.

(S3) Wang, J.; Cieplak, P.; Kollman, P. A. How well does a restrained electrostatic potential (RESP) model perform in calculating conformational energies of organic and biological molecules? *J. Comput. Chem.* **2000**, *21*, 1049–1074.

(S4) Jo, S.; Kim, T.; Iyer, V. G.; Im, W. CHARMM-GUI: a web-based graphical user interface for CHARMM. *J. Comput. Chem.* **2008**, *29*, 1859–1865.

(S5) Lee, J.; Hitzenberger, M.; Rieger, M.; Kern, N. R.; Zacharias, M.; Im, W. CHARMM-GUI supports the Amber force fields. *J. Chem. Phys.* **2020**, *153*.

(S6) Schmalhorst, P. S.; Deluweit, F.; Scherrers, R.; Heisenberg, C.-P.; Sikora, M. Overcoming the limitations of the MARTINI force field in simulations of polysaccharides. *J. Chem. Theory Comput.* **2017**, *13*, 5039–5053.

(S7) Benayad, Z.; von Bülow, S.; Stelzl, L.; Hummer, G. Simulation of FUS protein condensates with an adapted coarse-grained model. *J. Chem. Theory Comput.* **2020**, *17*, 525–537.
